# In Vitro Evaluation of Light-Induced Cytotoxic Property: Synergistic Effects of Anthocyanin/Curcumin as a Photosensitizer

**DOI:** 10.7759/cureus.48537

**Published:** 2023-11-08

**Authors:** Dhanya M, Umamaheswari TN, Rajalakshmanan Eeswaramoorthy

**Affiliations:** 1 Oral Medicine and Radiology, Saveetha Instituite of Medical and Technical Sciences, Chennai, IND; 2 Oral Medicine and Radiology, Saveetha Institute of Medical and Technical Sciences, Chennai, IND; 3 Biomaterials, Saveetha Institute of Medical and Technical Sciences, Chennai, IND

**Keywords:** photosensitizer, pdt, anthocyanin, curcumin, photodynamic therapy

## Abstract

Background: Photodynamic therapy is one of the non-invasive treatment modalities used to treat a wide range of oral mucosal lesions including oral leukoplakia, oral lichen planus, post-radiation-induced oral mucositis, and aphthous ulcerations. High-level laser therapy has also been employed in the management of oral squamous cell carcinomas (OSCCs). Photodynamic therapy includes three major components: photosensitizer, light source, and presence of oxygen. Examples of commonly used photosensitizers are methylene blue; porphyrins; and 5-aminolevulinic acid (5-ALA) with light sources of differing wavelengths such as diode, neon, and argon helium.

Methodology: Anthocyanin extract was prepared from 25 g of Punica granatum diluted with methanol through the flash evaporation method. Curcumin extract was prepared by diluting initially with dimethyl sulfoxide (DMSO) and later with distilled water. Post-preparation, the samples were treated with MCF-7 cells (cancer cell line) and the MTT (mono-tetrazolium salt) test was performed to determine cell viability.

Results: The anthocyanin group exhibited cell death of 27%, whereas the curcumin group exhibited cell death of 17%. A group containing a combination of anthocyanin-curcumin exhibited cell death of 30% compared to that of the control group proving to be superior among all the groups.

Conclusion: Anthocyanin-curcumin proved to have a light cytotoxic property and could be further used in managing various oral mucosal lesions after evaluation of other photophysical properties.

## Introduction

Oral potentially malignant disorders (OPMD) include oral leukoplakia, oral submucous fibrosis, and oral lichen planus which present with different clinical presentations [1]. The growth of OPMD is greatly influenced by tobacco usage [2]. Other etiological factors of OPMD commonly involve chewing betel nut and alcohol consumption. The overall prevalence of OPMD in the Indian population ranges from 13.2% to 13.9% [3]. The overall malignant transformation rate of OPMD groups is 7.9% [4].

Various treatment strategies have been advocated for OPMD such as preventive therapy consisting of lifestyle modifications followed by cessation of the predisposing habits [5]. Other management strategies included the use of topical and systemic corticosteroids, surgical management, cryotherapy, and the use of immunomodulators [6]. Photodynamic therapy is one of the emerging non-invasive treatment modalities of OPMD. It is based on the principle of singlet oxygen and free radicals production, leading to cell-specific destruction and membrane lysis of the target cells [7]. It is also suggestive that hyperproliferative cells in oral lichen planus and psoriasis undergo apoptotic effects [8].

Photosensitizers are substances that possess the ability to absorb light. The depth of penetration of the light source into the target tissue depends on the appropriate wavelength [9]. Among various photosensitizers, 5-aminolevulinic acid (5-ALA) serves as a biological precursor associated with the heme pathway and it also has a less prolonged effect of photosensitivity or cumulative toxicity [10]. Similar to 5-ALA, methylene blue belonging to the class of phenothiazinium has a wide range of advantages, namely better penetration when excited by a light source of 635 nanometers and the presence of antimicrobial properties [11]. In vitro studies using methylene blue prove to have the presence of every photophysical property [12].

Modern analytical tools make substances obtained from more accessible natural sources. Curcumin, an orange-yellow compound and a main composition of turmeric, can also be used as a photosensitizing agent as it has a photosensitive action against various bacteria, fungi, and other drug-resistant strains [13]. Punica granatum are flavonoids rich in anthocyanin that possess both antioxidant and anti-inflammatory properties. They exhibit bright colors such as purple and red [14]. The rationale of this current study was to determine whether the combination of curcumin and anthocyanin can have synergistic benefits to provide a better light cytotoxic profile.

Aim and objectives 

The aim of the study was to assess the cell viability in four groups, namely controls, anthocyanin, curcumin, and anthocyanin+ curcumin, in the presence of a light source (light-emitting diode (LED) source) of 635 nm. The objective was to compare the level of cell viability, thereby assessing the light toxicity among different groups (control, curcumin, anthocyanin, anthocyanin with curcumin).

## Materials and methods

Study setting and sample groups

**Figure 1:**
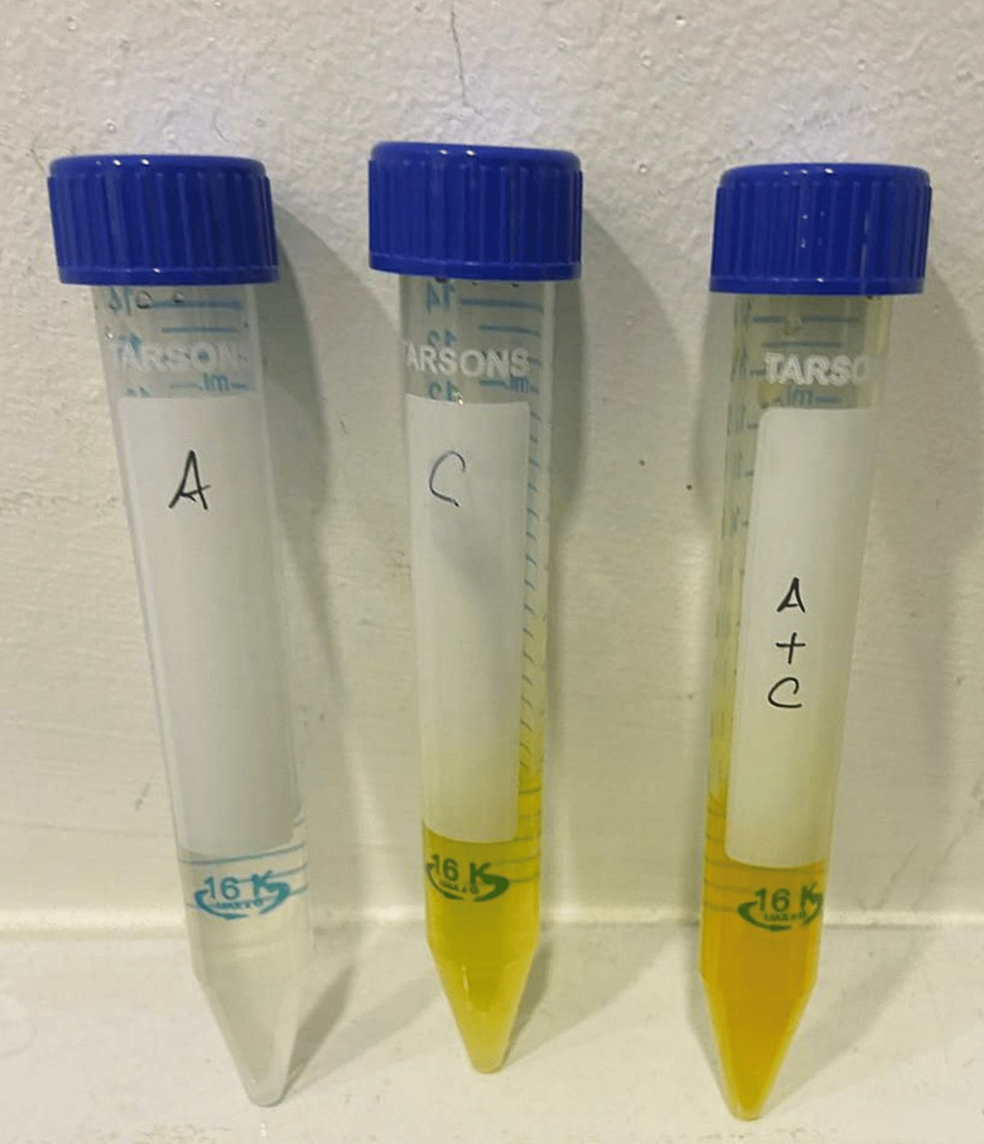
Sample groups Figure 1 depicts the samples of three experimental groups which was obtained through a series of extraction process and, furthermore, their concentrations were based on their individual molecular weights. Sample A denotes the anthocyanin compound, sample C denotes the curcumin compound, whereas sample A+C denotes the combination of anthocyanin and curcumin.

An in vitro study was conducted in the institute’s Centre for Molecular Medicine and Diagnostics. Since this was an in vitro study, G power sample size calculation was not applicable. The study consisted of four groups. Group 1 was the control, which consisted of only MCF-7 cells without incubation with prepared solutions, Group 2 consisted of 0.5 mL of anthocyanin post-flash evaporation diluted in 100 mL of water (Figure 1), Group 3 consisted of 0.5 mL of curcumin diluted in 100 mL of water (Figure 1), and Group 4 consisted of 0.25+0.25 mL of curcumin combined with anthocyanin in 100 mL of water (Figure 1).

Curcumin and anthocyanin extraction

**Figure 2:**
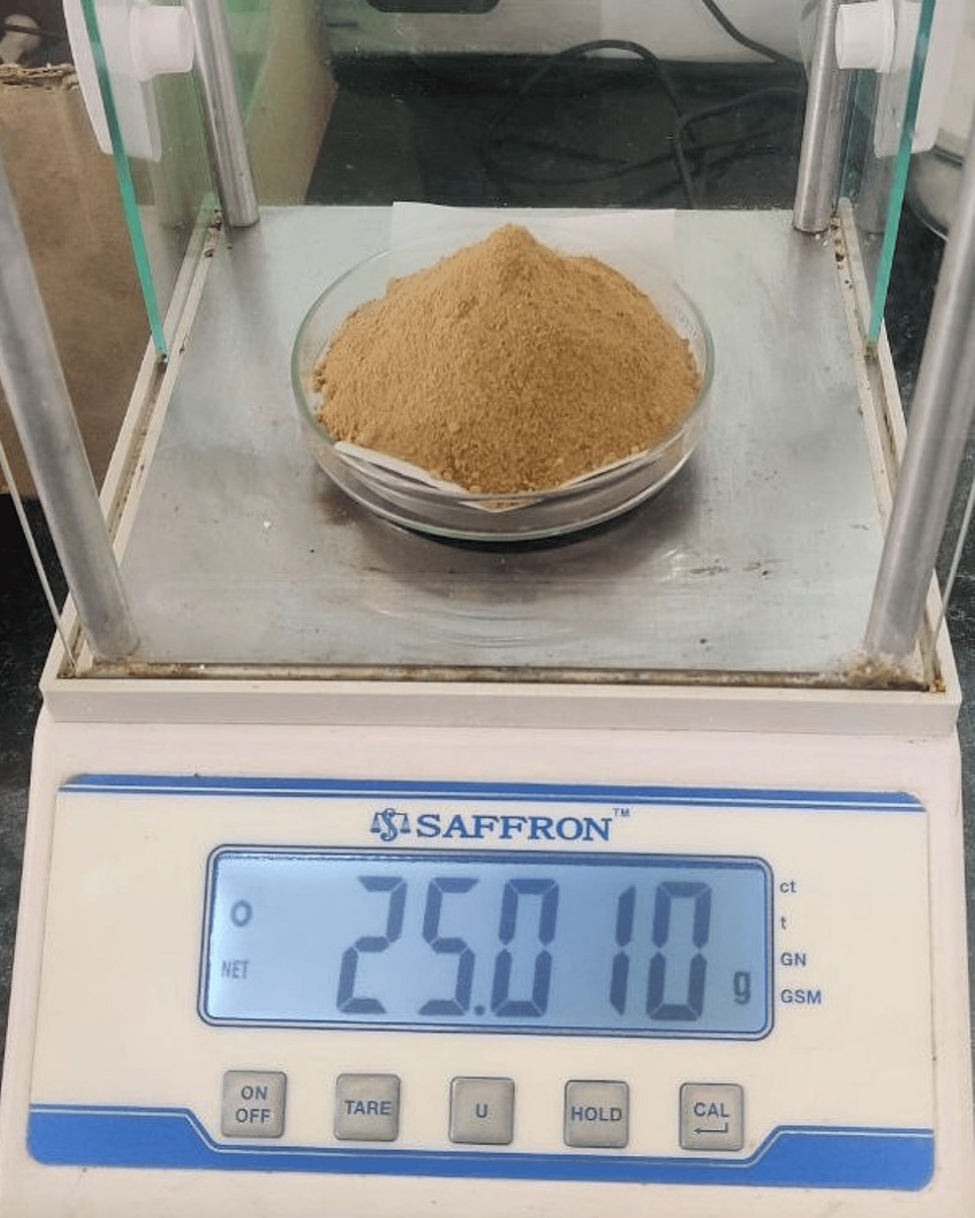
Grated Punica granatum Figure 2 depicts the raw component of anthocyanin from the peel powder of Punica granatum. Later, 25 g of dried and grated Punica granatum powder was weighed.

**Figure 3:**
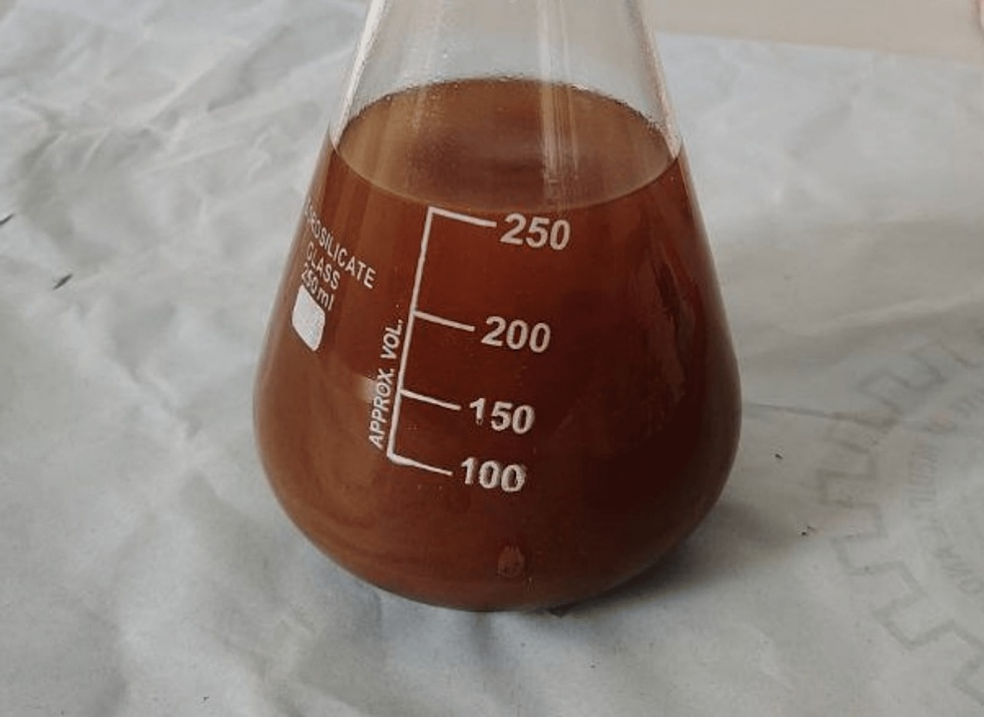
Dilution of Punica granatum mixture Figure 3 depicts the dilution of anthocyanin from the peel powder of Punica granatum with an organic solvent methanol in a 250 mL beaker.

**Figure 4:**
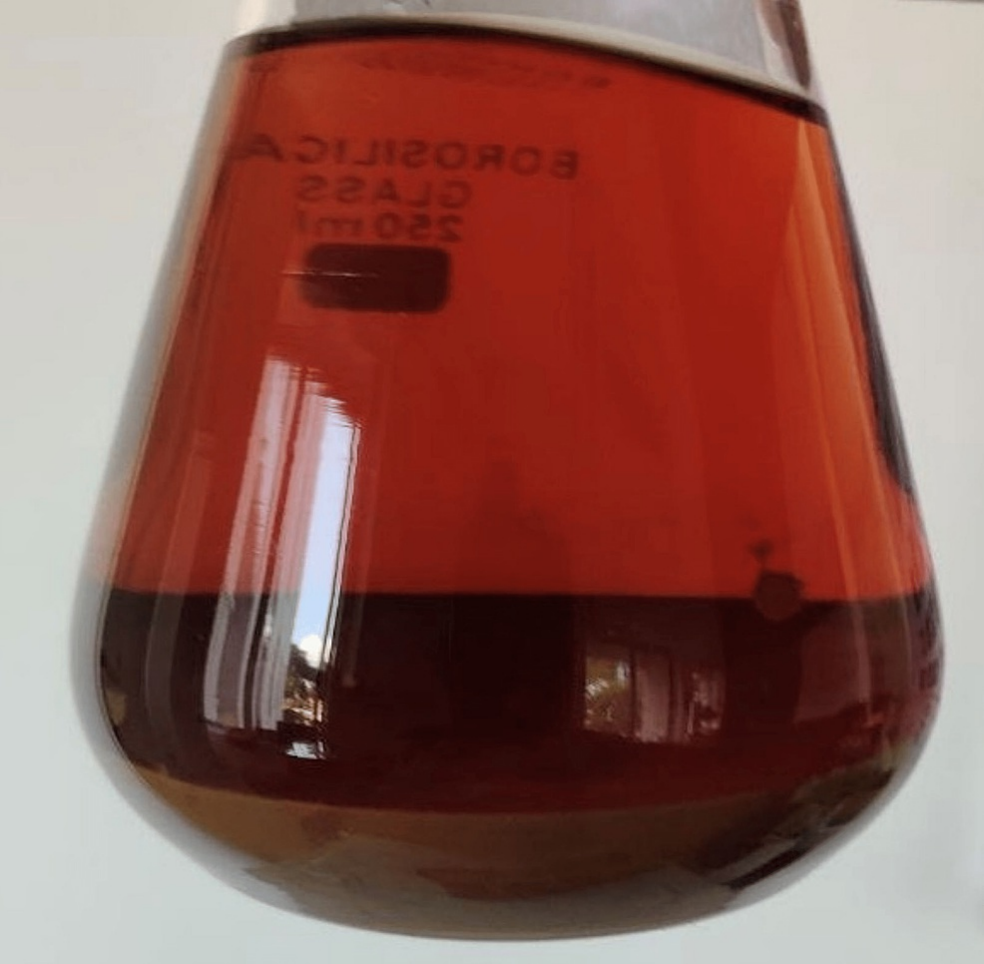
Sedimented form of anthocyanin Figure 4 depicts the sedimentation of the anthocyanin mixture at the bottom of the conical beaker after undergoing trituration for 24 hours. Later, the mixture was filtered and set up for flash evaporation.

**Figure 5:**
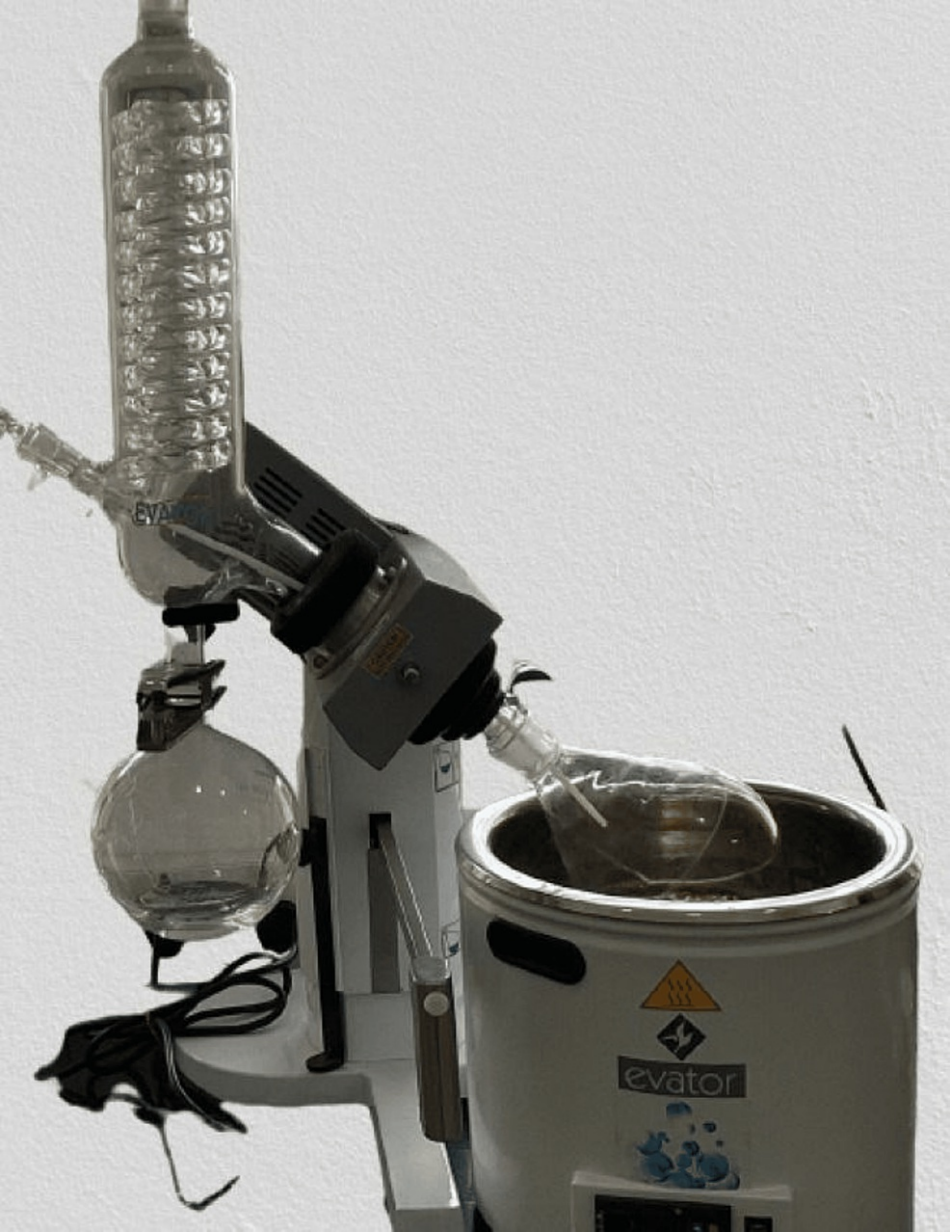
Flash evaporation of anthocyanin Figure 5 depicts the process of flash evaporation with the filtered form of anthocyanin processed at the temperature of 32°C. This process was done using reduced pressure and temperature by allowing the excess component to pass through a series of chambers with low pressure to be flashed repeatedly.

Curcumin was extracted from the compound curcuma longa species, and initially, curcumin was dissolved in 2 mL of dimethyl sulfoxide (DMSO) solution; then the dissolved form of curcumin was further diluted with distilled water. Anthocyanin was extracted from the peel powder of Punica granatum, which was grated and weighed (Figure 2). According to the extraction process of anthocyanin, an organic solvent methanol was used as a medium, and 25 g of peel powder was diluted with 250 mL of methanol (Figure 3); after trituration, the mixture of anthocyanin was filtered (Figure 4) and then the pure form was extracted using flash evaporation method at the temperature of 32°C (Figure 5).

Light toxicity and mono-tetrazolium salt assay

MCF-7 cells (cancer cell line) were utilized for the evaluation of the light toxicity profile. These cells were incubated in the prepared concentrates (curcumin, anthocyanin, and curcumin+anthocyanin) for half an hour. Once the incubation period was concluded, cells were illuminated with an LED source of 635 nm from which peak absorption was observed at 424.5 nm; later they were washed and assessed for cellular viability using the MTT viability test. The MTT comprises a quaternary tetrazole ring with a positive charge encompassing four nitrogen atoms and encircled by three aromatic rings. The core tetrazole ring is broken when the MTT is reduced which in turn also causes the creation of formazan, which has a water-insoluble violet-blue characteristic. MTT reagent could enter the membranes of the cell and mitochondria. This evaluation relied on the number of cells, metabolic activity of cells, amount of the reagent entering the cells, and timing of formazan crystals.

The spectrometric investigations were carried out using a wavelength of 635 nm, with a bandwidth of 1.0 nm, and a scan speed of 1000 nm/min. Those results showed a peak absorption at 424.5 nm. The 635 nm wavelength was employed to irradiate the cells in all three groups. Future clinical trials will be carried out with the anthocyanin+curcumin group with its peak absorbing wavelength.

## Results

**Figure 6:**
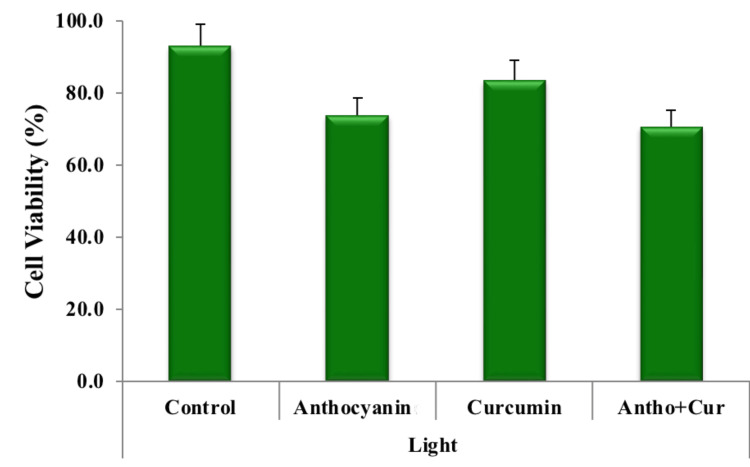
Graph depicting cell survival rate among control versus experimental groups

**Figure 7:**
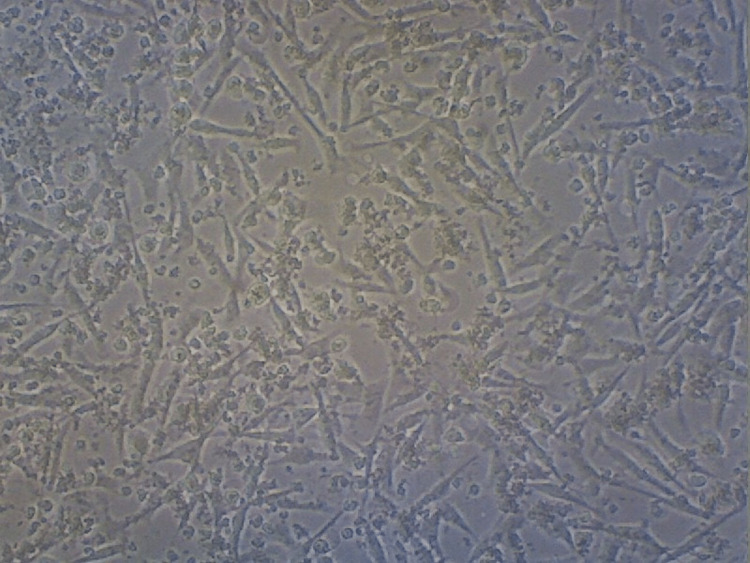
Cell viability - control group Depicts a dense cellular population indicative of more cellular viability (93%).

**Figure 8:**
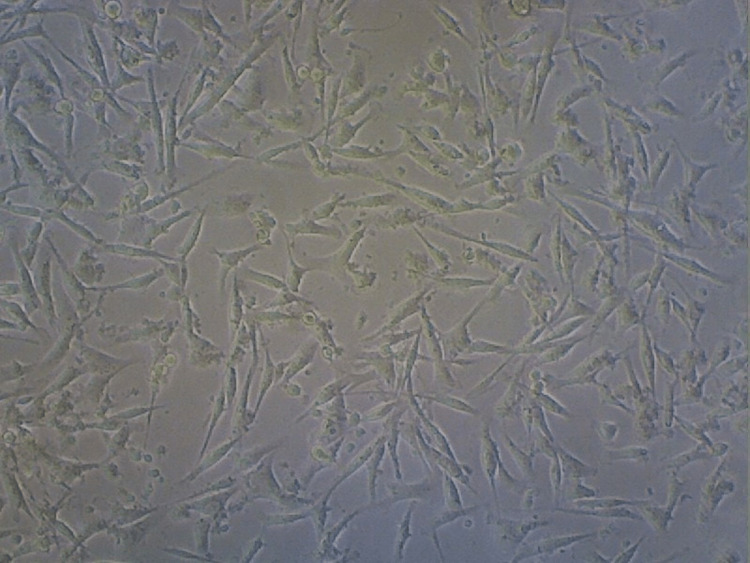
Cell viability - curcumin group Depicts a decrease in the cell number by 17% in the curcumin group from that of the control group, which indicates cell death.

**Figure 9:**
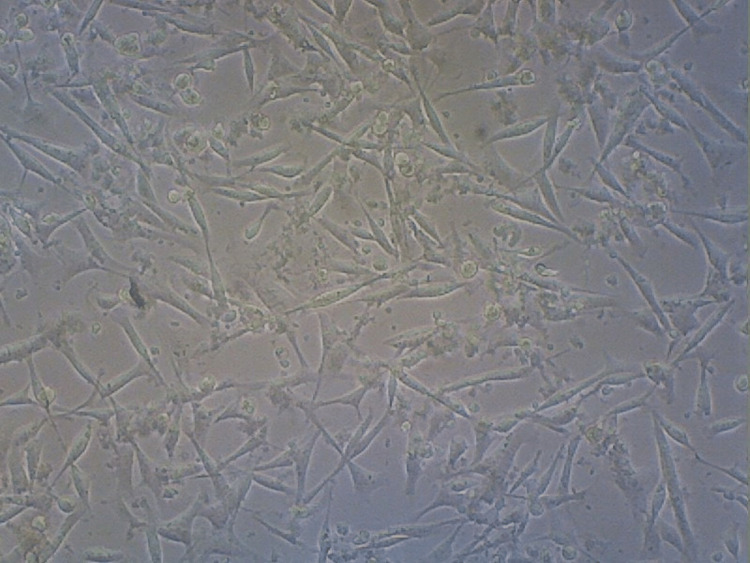
Cell viability - anthocyanin group Depicts a reduction in the number of cells by 27% in the anthocyanin group from that of the control group indicating cell death.

**Figure 10:**
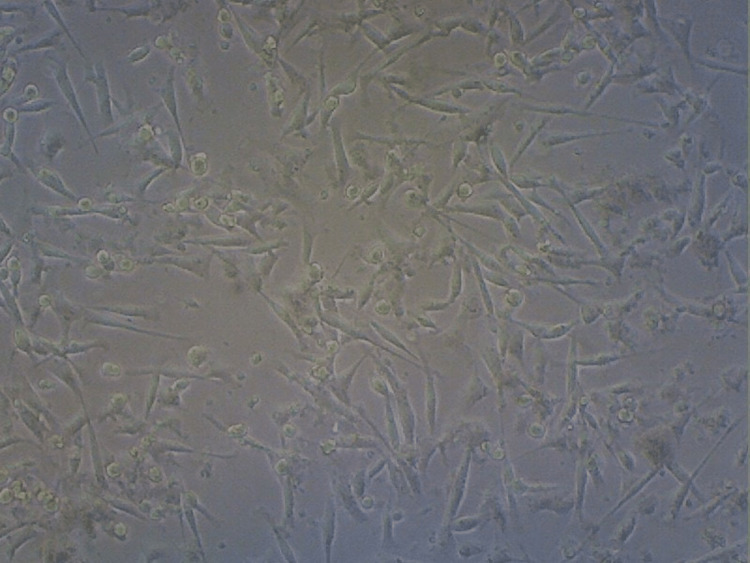
Cell viability - curcumin+anthocyanin group Depicts a reduction in the number of cells by 30% in the curcumin and anthocyanin group compared to that of the control group.

The percentage of cell viability in the groups was plotted in a graph (Figure 6). The results depict that the control group consisting of the cells had a viability percentage of 93% (Figure 7). Cell death of about 27% was observed in the anthocyanin group from that of the control group (Figure 8) and a reduction in cells of about 17% was noted in the curcumin group compared to that of the control group (Figure 9). Finally, there was a significant percentage of cell death of about 30% observed in the combination group consisting of anthocyanin and curcumin forecasting superior synergistic efficacy (Figure 10). These results were obtained through statistical analysis by following ANOVA, and the results obtained were significant to that of the control group.

## Discussion

OPMD have the potency to transform into oral squamous cell carcinoma (OSCC). One of the most prevalent precancerous lesions in the oral cavity is oral leukoplakia, which has a 20% chance of developing into OSCC. One of the management options employed in OPMD patients was photodynamic therapy [15]. Photodynamic therapy consists of different generations of photosensitizers causing photocytotoxic responses exclusively within the pathological tissues, pertaining to the region of photosensitizer distribution, allowing for selective destruction [16].

Photodynamic therapy employs the use of different types of photosensitizers with specific wavelengths in photodynamic therapy. A study by Mutafchieva et al. treated erosive and atrophic variants with laser treatment with low power (LLLT). They employed an 810 nm diode laser for 30 seconds in their approach [17]. Similarly, Wang et al. used a specialized device called a plum blossom needle to increase the incubation time of the photosensitizer in oral leukoplakia. The photosensitizer that they used was 10% ALA with a light source of 635 nm [18]. In this current study, we have used an LED source of 635 nm from which peak absorption was observed at 424.5 nm. The study by Tarasenko et al. used high-level lasers: Er:YAG and Nd:YAG of wavelengths 2940 nm and 1064 nm, respectively; this study was compared with that of scalpel surgery and its compliance [19]. The conventional photosensitizers employed in the previous studies reported adverse effects such as higher grades of pain, burning sensation, inflammation, and grade 3 transaminase elevations [20,21]. Whereas to overcome the adverse effects of conventional photosensitizers, herbal preparation of photosensitizer has been formulated using curcumin and anthocyanin in the present study.

Curcumin as a photosensitizer has been used in various studies such as the study by Leite et al. that assessed the effects of PDT with curcumin-aided photosensitizer mouthwash, which proved to have a significant decrease in the oral microflora, and the p-value was less than 0.05. The wavelength of the LED used was 455 nm [22]. According to the evaluation of the photophysical properties of curcumin by Kazantzis et al., curcumin proved to possess light cytotoxic property, dark cytotoxic property, UV absorption spectra, intracellular localization, reactive oxygen species (ROS) production, cell culture conditions, and photobleaching [23]. This is in correlation with the current study as curcumin and anthocyanin proved to have light cytotoxic properties. The study by Ma Jing et al. used curcumin as a photosensitizer in concentrations of 20 mm, 40 mm, 60 mm, 80 mm, and 100 mm in photodynamic therapy of oral candidiasis. Curcumin was used in this study as it had an increasing likelihood of various photophysical properties along with anti-microbial property, anti-inflammatory property, and anti-oxidant properties [24]. Anthocyanin as a photosensitizer has been used in a study by Spinei et al. against streptococcus species. Results showed a mean reduction in the colony-forming units when anthocyanin was combined with methylene blue with a P-value of <0.005 [25]. This proves that anthocyanin has a synergistic property and goes well with other natural compounds such as curcumin and riboflavins. 

Methodology and the mode of evaluation of the cytotoxicity play a vital role. A study by Dhanvanth et al. assessed the cytotoxicity of tulsi, aloe vera, and turmeric using enzyme-linked immunosorbent assay (ELISA) [26]. Similarly, a study by Dascalu et al. used mesenchymal stem cells from human dental pulp and keratinocyte cell lines from humans (HaCaT) to assess cell viability [27]. The MTT assay was utilized in this study with MCF-7 cells to determine the vitality of cells. For a particular photosensitizer to be effective, it should possess the required photophysical properties. In the current study, one of the photophysical property, the light cytotoxic property, was assessed and proved that the combination of anthocyanin and curcumin had better synergistic effects when compared with that of the single compounds, which also proved to reduce the adverse effects.

Limitations

Besides various therapeutic advantages, the current study failed to fulfill certain criteria that include the comparison with conventional photosensitizers such as toluidine blue and 5-ALA. The presence of other photophysical properties necessary for a conventional photosensitizer such as dark cytotoxicity, ROS production, and UV absorption spectra have not been evaluated in this study.

## Conclusions

In conclusion, the present study offers a promising alternative approach utilizing herbal photosensitizer in the management of potentially malignant disorders and oral cancers. The combination of this novel gel with curcumin and anthocyanin has proven to have a synergistic effect on the culture media. Future research could focus on the investigation of additional photophysical properties of this herbal gel, as efficient photosensitizers are likely to contain a number of unique qualities.
